# National Insurance Medical Benefit Act, 1913, and the Amendment Bill, 1920

**Published:** 1920-02-07

**Authors:** 


### NATIONAL INSURANCE MEDICAL BENEFIT ACT, 1913, AND THE AMENDMENT BILL, 1920.

The following has been sent to all Members of Parliament by the Federation of Medical and Allied Societies, 5 Vere Street, Cavendish Square, W. 1, whose Executive Council requests its careful consideration in view of its importance to the community as a whole:—

A Conference of representatives of medical, dental, nursing, pharmaceutical, and midwives' organisations, convened by the Federation of Medical and Allied Societies and held at Steinway Hall on December 12, 1919, adopted the following resolutions:—

"That in the opinion of this Conference of medical and allied organisations the draft Regulations for 1920, proposed to be made by the Minister of Health with respect to the administration of the National Health Insurance Medical Benefit, are not adapted to secure the most efficient service from the medical profession, nor adequate results to the community."

"That a public inquiry into the working of the medical benefit under the Acts be asked for to take evidence and report."

A deputation from the Federation met the Medical Committee of the House of Comons in conference, by invitation, on December 17, 1919, and obtained the support of that Committee in regard to its request for a public inquiry.

On January 23, 1920, a widely representative deputation, introduced by Sir Wm. Watson Cheyne, Bart., K.C.M.G., C.B., M.P., was received by the Minister of Health, which placed before him its reasons for asking for an immediate public inquiry.

The Minister, in a sympathetic but wholly inconclusive reply, stated that he had himself condemned the lamentable chaos of our present health services; that he did not want to burke an inquiry, and that if such an inquiry as the deputation asked for were the best way of instructing the public and the medical profession, it would serve a great purpose. He held, however, that he must await the report of his Medical Consultative Council, and if he became convinced that any advantage could be gained by a public inquiry he would not hesitate to recommend it.

The Federation is informed that under the limitations imposed by the present Act it is impossible to utilise the lessons of the war on health questions, and that it is therefore impossible to have a really efficient service under its provisions.

It suggests that the Consultative Council should have the advantage of an inquiry into the working of the Act during the past seven years, in order to ascertain in what way the present system is defective and how it can be brought into line with modern knowledge, before it produces suggestions for a new and probably costly scheme of medical services for the country.

The Consultative Council cannot itself hold a public inquiry for the taking of such evidence.

The Federation therefore asks you to support a demand for an immediate public inquiry before any expansion or alterations in the provisions of the Insurance Act is proposed.

N. Howard Mummery, M.R.C.S., L.R.C.P.,
February 4, 1920. General Secretary.

